# Cafeteria diet induces global and *Slc27a3*-specific hypomethylation in male Wistar rats

**DOI:** 10.1080/21623945.2021.1886697

**Published:** 2021-02-11

**Authors:** Amsha Viraragavan, Tarryn Willmer, Oelfah Patel, Albertus Basson, Rabia Johnson, Carmen Pheiffer

**Affiliations:** a Biomedical Research and Innovation Platform, South African Medical Research Council, Tygerberg, South Africa; b Department of Biochemistry and Microbiology, University of Zululand, Kwa-Dlangezwa, South Africa; c Division of Medical Physiology, Faculty of Health Sciences, Stellenbosch University, Tygerberg, South Africa; d Division of Clinical Pharmacology, Department of Medicine, Faculty of Medicine and Health Sciences, University of Stellenbosch, Tygerberg, South Africa

**Keywords:** Obesity, insulin resistance, Slc27a3, DNA methylation, adipose tissue, visceral, subcutaneous

## Abstract

Increased visceral adipose tissue (VAT) is associated with metabolic dysfunction, while subcutaneous adipose tissue (SAT) is considered protective. The mechanisms underlying these differences are not fully elucidated. This study aimed to investigate molecular differences in VAT and SAT of male Wistar rats fed a cafeteria diet (CD) or a standard rodent diet (STD) for three months. The expression of fatty acid metabolism genes was analysed by quantitative real-time PCR. Global and gene-specific DNA methylation was quantified using the Imprint® Methylated DNA Quantification Kit and pyrosequencing, respectively. Bodyweight, retroperitoneal fat mass, insulin resistance, leptin and triglyceride concentrations and adipocyte hypertrophy were higher in CD- compared to STD-fed rats. The expression of solute carrier family 27 member 3 (Slc27a3), a fatty acid transporter, was 9.6-fold higher in VAT and 6.3-fold lower in SAT of CD- versus STD-fed rats. Taqman probes confirmed increased Slc27a3 expression, while pyrosequencing showed Slc27a3 hypomethylation in VAT of CD- compared to STD-fed rats. The CD decreased global methylation in both VAT and SAT, although no depot differences were observed. Dysregulated fatty acid influx in VAT, in response to a CD, provides insight into the mechanisms underlying depot-differences in adipose tissue expansion during obesity and metabolic disease.

**Abbreviations:** CD: cafeteria diet; E2F1: E2F Transcription Factor 1; EMSA: electrophoretic mobility shift assay; EGFR: epidermal growth factor receptor; GCF: GC-Rich Sequence DNA-Binding Factor; HOMA-IR: Homeostasis model for insulin resistance; NKX2-1: NK2 homeobox 1; PCR: Polymerase chain reaction; qRT-PCR: quantitative real-time PCR; RF: retroperitoneal fat; SAT: subcutaneous adipose tissue; Slc27a3: solute carrier family 27 member 3; STD: standard diet; TNFα: tumour necrosis factor alpha; TTS: transcriptional start site; T2D: Type 2 Diabetes; VAT: visceral adipose tissue; WT1 I: Wilms’ tumour protein 1

## Introduction

The chronic consumption of high caloric foods leads to obesity and contributes to the development of type 2 diabetes and cardiovascular disease [1]. White adipose tissue (WAT) is a key endocrine organ that plays an active role in maintaining metabolic homeostasis in response to nutritional and environmental cues by altering lipid storage, tissue expansion through hypertrophy and/or hyperplasia and secretion of adipokines [2]. During excessive weight gain, the expansion and remodelling of WAT renders the tissue dysfunctional, leading to insulin resistance and metabolic derangement [2]. Studies in humans and rodents have shown that fat stored in visceral WAT depots is strongly linked to metabolic derangements and is associated with increased risk for obesity-associated comorbidities, possibly owing to its lipolytic nature and contribution to chronic low-grade inflammation [3,4]. Conversely, accumulation of subcutaneous WAT appears to be protective against metabolic diseases as it is associated with a reduced incidence of type 2 diabetes and cardiovascular disease when adiposity is comparable [5,6]. However, the molecular mechanisms underlying the depot differences in disease risk remain elusive.

Epigenetic processes may contribute to the development of obesity by mediating the effects of environmental exposures on gene expression and energy homeostasis [7,8]. DNA methylation is the most extensively studied epigenetic modification and occurs by covalent addition of a methyl group at the 5^th^ carbon of the cytosine ring, resulting in 5-methylcytosine [9]. Studies have shown that changes in nutrient cues, such as the consumption of high fat, high sugar diets, differentially modulate DNA methylation of key metabolic genes [10,11].

Previously we showed that a high fat, high sugar cafeteria diet (CD) induced obesity and concomitant gene expression changes in the liver and muscle from male Wistar rats [12]. In the present study, we aimed to investigate whether CD induces depot-specific gene expression and DNA methylation changes in subcutaneous adipose tissue (SAT) and retroperitoneal fat, a marker of visceral adipose tissue (VAT). Here we show that CD exposure evoked depot-specific upregulation of the *Slc27a3* long chain fatty acid transporter, which was accompanied by both global and *Slc27a3*-specific DNA hypomethylation. These findings provide insights into the physiological differences between VAT and SAT depots and suggest that differences in fatty acid transport may underly the distinct adipose tissue expansion properties observed between these depots.

## Methods

### Animals

Details of the study design have been described previously [12]. Briefly, three-week old male Wistar rats were fed either a high fat, high sugar cafeteria diet (CD) (n = 10) consisting of a patty containing 40% fat, 15% protein and 44% carbohydrates and a jelly cube supplemented with 15% (w/v) sucrose and fructose or a standard diet (STD) (n = 10) consisting of 11% fat, 15% protein and 74% carbohydrates for three months. Water was given *ad libitum*. SAT and retroperitoneal fat, a marker of VAT, were subsequently harvested for histological, gene expression and DNA methylation analysis. The selection of retroperitoneal fat as a representative of VAT is based on reports that (1) lipids from this depot drain directly into the kidney, pancreas and vena cava and (2) retroperitoneal fat mass in humans is directly associated with metabolic syndrome and hypertension [13]. Ethics for this study was granted (ECRA # 11/03/B) by the Ethics Committee for Research on Animals (ECRA) of the South African Medical Research Council (SAMRC, Tygerberg, South Africa).

### Histological analysis

SAT and VAT sections were prepared by paraffin embedding 4 µm thick sections overnight in 4% paraformaldehyde. Tissues were then mounted on microscope glass slides and dried overnight at 37 °C. Dried slides were stained with haematoxylin and eosin (H&E) as described by Fischer et al. [14]. Digital images were captured using a Nikon Eclipse Ti inverted microscope (Tokyo, Japan) at 20× magnification for a minimum of nine rats per group. Adipocyte number, diameter and area were measured in ImageJ for a total of four fields per rat.

### DNA isolation

Genomic DNA from approximately 100 mg SAT and VAT was isolated using the Qiagen DNeasy Blood & Tissue Kit (Qiagen, Valencia, USA) as per the manufacturer’s instructions. DNA purity and quantity were assessed using the Nanodrop Spectrophotometer (Nanodrop™ One, Thermo Fisher Scientific, Waltham, USA) and Qubit fluorometer (Life Technologies, CA, USA), respectively.

### Global DNA methylation

Global DNA methylation was analysed using the Imprint® Methylated DNA Quantification kit (MDQ1, Sigma-Aldrich, St. Louis, USA) according to the manufacturer’s instructions, as previously described [15]. Briefly, 100 ng genomic DNA was bound to the ELISA plate, after which the methylated DNA fraction was detected using a 5-methylcytosine monoclonal antibody and the absorbance was measured on a SpectraMax i3 plate reader (Molecular Devices®) at 450 nm. The percent global DNA methylation was calculated relative to the methylated DNA control, which was included in the kit. All samples were analysed in duplicate.

### Pyrosequencing

The PyroMark® Q96 ID Pyrosequencing System (Qiagen, Seoul, South Korea) was used to determine the methylation status of six CpG sites within *Slc27a3* which was differentially expressed in VAT. *Slc27a3* pyrosequencing primers (Rn_Slc27a3_01_PM PyroMark CpG assay) were pre-designed and purchased from Qiagen (Hilden, Germany). Prior to pyrosequencing, quantity and quality of amplicons were assessed by agarose gel electrophoresis. Visceral (100 µg) adipose samples were bisulphite converted using the EpiTect DNA kit (Qiagen, Hilden, Germany) as per the manufacturer’s recommendations. A thermal cycler was used to convert the DNA with two cycles of denaturation at 95°C for 5 minutes and incubation at 60°C for 10 minutes, followed by column-based DNA clean-up. PCR of 25 ng bisulphite converted DNA was carried out using the PyroMark PCR Kit (Qiagen, Valencia, CA, USA) under the following PCR conditions: 95°C for 15 minutes; 45 x (95°C for 30 seconds; 56°C for 30 seconds; 72°C for 30 seconds) and 72°C for 5 minutes. PCR reactions were carried out in a Veriti 96-well Thermal Cycler (Thermo Fisher Scientific, Waltham, USA) and the quality and size of amplicons were assessed by agarose gel electrophoresis. Pyrosequencing was carried out using the PyroMark Gold Q96 Reagents (Qiagen, Valencia, USA) on a PyroMark ID pyrosequencer (Qiagen, Seoul, South Korea), and the methylation level was calculated using the PyroMark Q96 software (Version 1.0.10; Qiagen) program. All pyrosequencing assays were validated using different ratios of methylated:unmethylated bisulphite converted DNA (0, 10, 25, 50, 75, 90 and 100%) (Qiagen, Valencia, CA, USA), from which standard curves were constructed to determine primer sensitivity (Figure S1). Each pyrosequencing run contained no template negative controls and bisulphite conversion controls were incorporated within each assay sequence to assess conversion efficiency.

### RNA isolation

Total ribonucleic acid (RNA) from ~ 100 mg SAT and VAT was isolated using Qiazol (Qiagen, Maryland, USA) and purified using the RNeasy Mini Kit (Qiagen Maryland, USA) according to the manufacturer’s instructions. RNA purity and quantity were analysed on the Nanodrop spectrophotometer (Nanodrop™ One, Thermo Fisher Scientific, Waltham, USA). Quality was measured by running RNA samples on the Agilent 2100 Bioanalyzer (Agilent Technologies).

### Rat Fatty Acid Metabolism PCR array

A Rat Fatty Acid Metabolism RT^2^ Profiler PCR Array (Qiagen, Hilden, Germany) was used to screen the expression of 84 genes involved in regulating fat metabolism within SAT (n = 8) and VAT (n = 8) samples from rats fed a CD or STD diet. Complementary DNA (cDNA) was synthesized from 1 μg of total RNA using a commercial RT^2^ First Strand Kit (Qiagen, Hilden, Germany) according to manufacturer instructions. The cDNAs were then mixed with RT^2^ qPCR ROX master mix containing SYBR Green (Qiagen, Hilden, Germany) and thereafter pipetted into respective wells of the RT^2^ Profiler PCR Array plate. The plate was sealed with a MicroAmp optical adhesive film (Thermo Fisher Scientific, Waltham, USA) and centrifuged for 1 minute at 1000 x g at room temperature. PCR was conducted on the Quantstudio 7™ Flex Real-Time PCR System (Applied Biosystems) with the following conditions: denaturation for 15 min at 95°C, combined annealing and extension for 40 cycles at 60°C for 1 min. Default baseline and cycle threshold (Ct) settings were used and the PCR array values were exported to an excel file to create a table of Ct values, which was uploaded onto the data analysis web portal (http://www.qiagen.com/geneglobe). Gene expression was quantified using the ΔΔCt method and normalized to Beta-2-microglobulin (B2m) and ribosomal protein lateral stalk subunit P1 (Rplp1) housekeeping genes. PCR reports were generated after removing samples with multiple melting curves or those with a Ct value above 40. Only genes that were differentially expressed in VAT and SAT in the CD compared to STD-fed rats with a p-value <0.05 were considered for further analysis.

### qRT-PCR validation with Taqman probes

Differentially expressed genes identified from the PCR arrays were further assessed by qRT-PCR using Taqman probes. Complementary DNA was generated by reverse transcribing a total of 20 ng RNA using the High Capacity Reverse Transcription kit (Life Technologies, CA, USA) following instructions from the manufacturer. Taqman gene expression probes and TaqMan Universal PCR Master Mix (Thermo Fisher Scientific, Waltham, USA) were used to measure the transcriptional status of *Slc27a3* (Rn01455596_m1, Thermo Fisher Scientific, Waltham, USA) in SAT and VAT from STD and CD-fed rats. Relative expression levels were calculated using the standard curve method. B2m (Rn00560865_m1, Thermo Fisher Scientific, Waltham, USA) and Rplp1 (Rn03467157_gH, Thermo Fisher Scientific, Waltham, USA) were selected as the two best endogenous controls from the PCR array using Norm finder [16]. The selected probes spans exons, and thus, RNA did not require DNAse-treatment.

### In silico mapping of putative transcription factor binding sites

*In silico* analysis was performed to identify potential transcription factors that bind the regions spanning the six *Slc27a3* CpG sites investigated in this study. The *Slc27a3* gene sequence containing the CpG island of interest was obtained from the UCSC Genome Browser (https://genome.ucsc.edu/- Rat 5.0/rn5 assembly, accessed May 2020). The transcription factor prediction software PROMO [17] was used to identify the putative transcription factors within this region.

### Statistical analysis

Data presented in this study were captured in Microsoft Excel (Microsoft Office 2016) and statistical analysis conducted in GraphPad Prism software (Graphpad Prism® version 6.01, GraphPad Software, La Jolla, USA). Data are represented by the mean ± standard error of the mean (SEM). All data were normally distributed, as determined by the Shapiro-Wilk test and thus the unpaired Student’s t-test was used to test for significant differences between STD and CD groups. P ≤ 0.05 were considered statistically significant. A one-way analysis of variance for the *Slc27a3* pyrosequencing was performed to calculate statistical significance of methylation between the two diet groups and at which CpG site methylation differed in CD vs STD fed rats.

## Results

### Animal characteristics

The characteristics of animals used in this study have been reported previously (Table 1) [12]. After three months of diet exposure, the CD-fed rats were 20% (p < 0.001) heavier than STD-fed rats. Moreover, the retroperitoneal fat of CD-fed rats weighed 14 g more than that of STD-fed rats (p < 0.001). While no differences in blood glucose concentrations were observed between the groups, the CD-fed rats exhibited increased serum triglycerides (1.20 ± 0.10 vs 0.50 ± 0.10 mM, p < 0.01), fasting insulin concentrations (6.14 ± 2.79 vs 3.78 ± 1.97 ng/ml, p = 0.042) and homeostatic model assessment of insulin resistance (HOMA-IR, 1.65 ± 0.76 vs 1.00 ± 0.53, p = 0.039) compared with control rats. Consistent with these observations, circulating leptin levels were increased in CD-fed compared to STD-fed rats (Figure S2A, 24.81 ± 0.77 vs 4.91 ± 0.28 ng/ml, p < 0.001), whilst no differences were observed in serum tumour necrosis factor α (TNFα) levels (Figure S2B).

**Table 1.** Metabolic characteristics of CD- and STD-fed rats

Variable	STD (n = 10)	CD (n = 10)	P-value
Body weight (g)	474.00 ± 33.30	567.50 ± 27.86	p < 0.001
RF mass (g)	0.81 ± 0.06	2.79 ± 0.14	p < 0.001
Fasting glucose (mmol/L)	5.94 ± 0.43	5.98 ± 0.51	p = 0.850
Fasting insulin (ng/ml)	3.78 ± 1.97	6.14 ± 2.79	p = 0.040
HOMA-IR	1.00 ± 0.53	1.65 ± 0.76	p = 0.040
Triglycerides (mM)	0.50 ± 0.10	1.20 ± 0.10	p < 0.010

Abbreviations: HOMA-IR, Homeostasis model for insulin resistance; RF, retroperitoneal fat. Abbreviations: HOMA-IR, Homeostasis model for insulin resistance; RF, retroperitoneal fat.

### Increased hypertrophy in CD-fed rats

We next explored morphological differences in SAT and VAT depots in response to CD-feeding. We measured adipocyte area in H&E stained SAT and VAT harvested from rats fed either the CD or STD (Figure S3A). Results showed that three months of CD feeding resulted in a significant increase in adipocyte area within VAT, and although an increase in SAT adipocyte area was also observed (Figure S3A), these changes were not significant (Figure S3B). These findings suggest that CD-induced hypertrophic adipocyte expansion, which was more pronounced in VAT compared to SAT.

### Increased expression of Slc27a3 fatty acid transporter in VAT

**Figure 1.**
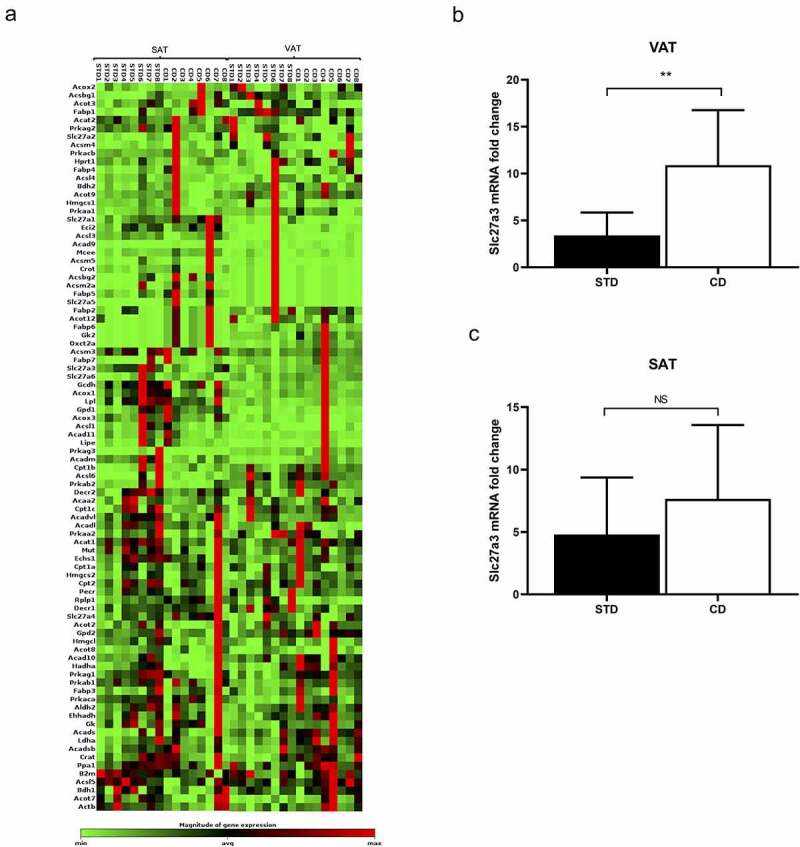
(a) Heatmap of up- and down-regulated genes, and those with unchanged expression in SAT and VAT of CD and STD rats as determined by the Rat Fatty Acid Metabolism RT^2^ Profiler PCR Array. Data are presented as mean ± SEM (n = 8). Taqman gene expression validation of *Slc27a3* in (b) VAT and (c) SAT of CD and STD rats. Data are presented as mean ± SEM (n = 10)

In order to gain deeper insights into the molecular differences between SAT and VAT, we next compared the gene expression profiles of these depots upon exposure to either CD or STD using commercially available PCR arrays. These arrays contain primers to 84 genes involved in fatty acid biosynthesis, transport and metabolism. The results revealed altered expression of four and ten genes in SAT and VAT, respectively in CD-fed rats compared to STD-fed rats (Table 2, Figure 1(a)). None of the genes remained significant after Bonferroni correction. Among the differentially expressed genes (p < 0.05), altered expression of one gene, solute carrier family 27 member 3 (*Slc27a3*), was common between both depots, where it was up-regulated by 9.6-fold in VAT (p = 0.019) and down-regulated by 6.3-fold in SAT (p = 0.048) from CD-fed rats compared to controls. Since Slc27a3 has previously been implicated in fatty acid transport [18,19], we speculated that the opposing expression patterns of this fatty acid handling protein in SAT and VAT may account for the differences in adipose tissue expansion properties observed between these depots. We therefore focused subsequent analysis on this gene.

**Table 2.** List of genes and their fold regulation assessed using a Rat Fatty Acid Metabolism PCR array in VAT and SAT

Gene	VAT	p-value	SAT	p-value
*Acot7*	14.0	**0.002**	−8.0	0.592
*Acox3*	3.2	**0.036**	−1.1	0.851
*Acsl1*	4.2	**0.016**	−4.5	0.310
*Bdh2*	5.7	**0.005**	−1.9	0.568
*Decr1*	2.4	**0.038**	1.4	0.390
*Lpl*	2.1	**0.034**	−1.1	0.538
*Ppa1*	2.9	**0.038**	−1.7	0.707
*Prkag1*	7.7	**0.012**	−4.7	0.273
*Slc27a3*	9.6	**0.019**	−6.3	**0.048**
*Acsbg1*	−5.0	**0.037**	7.4	0.177
*Acadm*	2.6	0.217	−10.2	**0.036**
*Acsl5*	1.8	0.254	−5.8	**0.045**
*Slc27a1*	2.0	0.311	2.2	**0.045**

Fold change refers to gene expression in CD-fed rats compared to STD-fed rats.

Abbreviations: VAT, visceral adipose tissue, SAT, subcutaneous adipose tissue

P-values in bold indicate statistical significance.

qRT-PCR analysis using Taqman probes confirmed that CD exposure led to a significant upregulation of *Slc27a3* messenger ribonucleic acid (mRNA) (p = 0.003) in VAT (Figure 1(b)), however, in contrast to the PCR array findings, no significant differences in *Slc27a3* expression was observed in SAT from CD-fed compared STD-fed rats (p = 0.236) (Figure 1(c)).

### Global and gene-specific DNA hypomethylation in CD-fed rats

**Figure 2.**
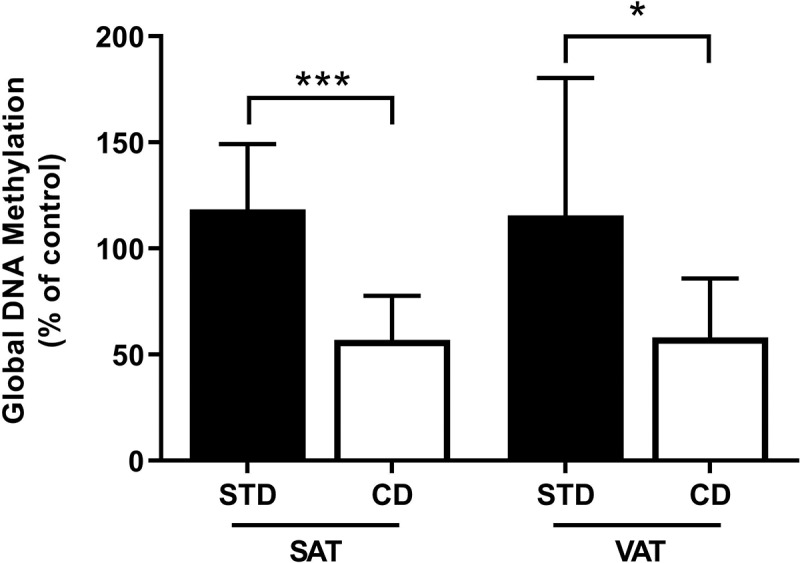
Global DNA methylation levels in SAT and VAT of rats fed a CD and STD. Methylation was calculated relative to the methylated DNA control (50 ng/mL). Data are presented as mean ± SEM (n = 10)

**Figure 3.**
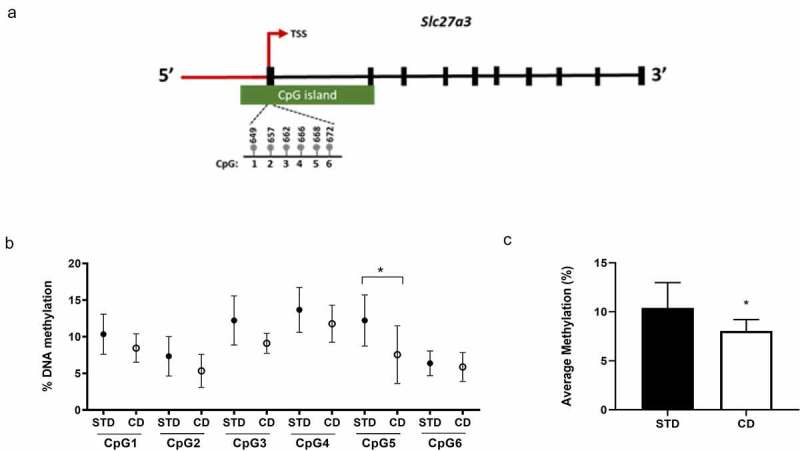
(a) Location of CpG sites investigated in *Slc27a3* proximal promoter and exon 1 region. (b) CpG site-specific and (c) mean methylation levels of *Slc27a3* in VAT of CD rats versus STD rats. Data are presented with mean ± SEM (n = 10)

To gain insight into the involvement of DNA methylation in the CD-induced gene expression changes in SAT and VAT, we assessed global DNA methylation levels using an enzyme-linked immunosorbent assay. Results revealed that DNA methylation levels were reduced by 50% in SAT (p < 0.001) and 52% in VAT (p = 0.025) from CD- compared to STD-fed rats (Figure 2). Since *Slc27a3* gene expression was significantly upregulated in response to CD-feeding in VAT, we next explored whether *Slc27a3* expression changes in this depot were due to DNA methylation. To this end, we analysed the DNA methylation status of the *Slc27a3* exon 1 region in VAT isolated from CD- or STD-fed rats using pyrosequencing. An analysis of six CpG sites situated within the first exon of *Slc27a3* (Figure 3(a)) revealed hypomethylation at one CpG site (CpG5), positioned 668 base pairs (bp) downstream from the transcriptional start site (TSS) (Figure 3(b)). Moreover, analysis of the cumulative methylation across all six CpG sites revealed significantly lower DNA methylation in CD- compared to STD-fed rats (Figure 3(c)). Since *Slc27a3* was not differentially expressed between the diet groups in SAT, we did not pursue pyrosequencing analysis for this depot.

### Identification of transcription factor binding sites

**Figure 4.**
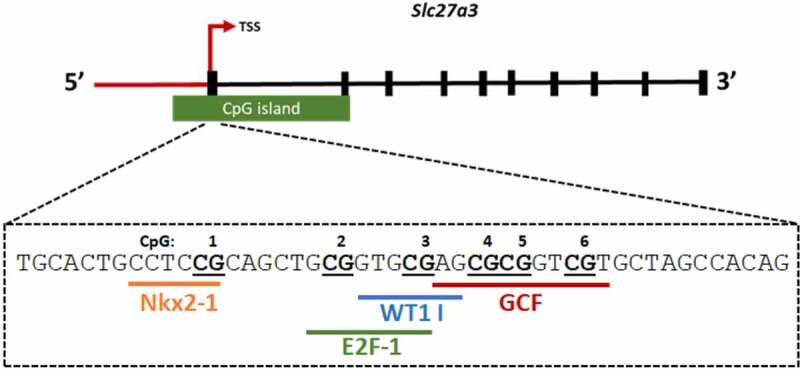
Schematic illustration showing predicted transcription factors to the *Slc27a3* region investigated by pyrosequencing. The *Slc27a3* gene contains a 850bp CpG island that extends from −176 bp upstream of the transcription start site to exon 2. Transcription factor binding sites spanning CpGs 1–6 (bold, underlined) within *Slc27a3* exon 1 were identified using PROMO software. The following factors were identified: Nkx2-1- NK2 homeobox 1, E2F-1 – E2F Transcription Factor 1, WT1 I – Wilms’ tumour protein 1 and GCF – GC-Rich Sequence DNA-Binding Factor

To explore the functional significance of the investigated CpG sites, *in silico* analysis was conducted to identify potential transcription factors that bind these regions. Transcription factor binding site prediction identified putative binding sites for NK2 homeobox 1 (Nkx2-1), E2F Transcription Factor 1 (E2F-1), Wilms’ tumour protein 1 (WT1 I) and GC-Rich Sequence DNA-Binding Factor (GCF) (Figure 4).

## Discussion

The current study aimed to explore DNA methylation and gene expression patterns in SAT and VAT during the early stages of diet-induced obesity in Wistar rats, in order to provide potential mechanisms by which these two adipose depots differ with regards to their expansion capabilities and consequently, pathophysiological roles in metabolic disease. Here we show that consumption of a high fat, high sugar CD led to significant increases in body weight, retroperitoneal fat mass, and serum insulin, triglyceride and leptin levels, as well as histological changes in adipocyte size. Furthermore, CD-exposure induced global DNA hypomethylation in both SAT and VAT, as well as VAT-specific changes in the expression of long chain fatty acid metabolism-related genes. Among these, a fatty acid transporter, *Slc27a3* was significantly upregulated in VAT, whilst its expression remained unaffected by CD in SAT. We further show that *Slc27a3* expression may be epigenetically upregulated in VAT, as DNA methylation at one CpG site within exon 1, proximal to the TSS, was significantly reduced in CD-fed rats compared to STD controls.

The endocrine functions of adipocytes are largely influenced by their size, with larger adipocytes being associated with insulin insensitivity, inflammation and metabolic dysfunction [20]. In the current study, we observed that VAT from CD-fed rats contained a greater percentage of larger adipocytes compared to SAT, which was accompanied by elevated serum insulin and triglycerides and increased expression of leptin, a pro-inflammatory cytokine belonging to the IL-6 family [21]. These findings are in line with other studies using rodent models of diet-induced obesity in [22–25]. Interestingly, in contrast to previous studies, we did not observe increased levels of the pro-inflammatory cytokine, TNFα, in response to CD feeding. This may be due to [1] different ages of the rats at the start of diet treatment (e.g. six weeks in [26] versus three weeks in this study) [2], differences in energy density and composition of the diets used (e.g. a high fat diet with a supra-physiological fat content of 60% kcal [27] versus a more physiological diet with a fat content of 40%, as is comparable to human diets in this study) and [3] the duration of high caloric diet feeding (e.g. 24 weeks in [28] versus 12 weeks in this study).

The increased deposition of fatty acids in adipose and other tissues is considered a hallmark of obesity. In the past few years, it has been established that fatty acids cross the plasma membrane via a protein-mediated mechanism involving one or more fatty acid-handling proteins, including a family of fatty acid transporters [18]. In the present study, we identified several differentially expressed genes involved in fatty acid transport and metabolism in SAT versus VAT in response to CD feeding. Slc27a3 is a membrane protein known for its critical role in fatty acid uptake by endothelial cells [18,19]. However, the role of Slc27a3 in obesity, if any, remains unclear. We note that studies have reported elevated *Slc27a3* expression in skeletal and cardiac muscle from mice fed a high-fat diet [29] and in H9c2 cardiomyocytes exposed to high glucose [30], and although the expression status of *Slc27a3* in adipose tissue is not well documented, Bouwman (2013) reported a significant downregulation of *Slc27a3* in human abdominal adipose tissue biopsies in response to a 5-week, calorie restriction weight-loss programme [31]. The authors speculated that the reduction in adipose tissue fatty acid import may, in part, explain the benefits of weight loss in reducing metabolic risk. This study, together with ours, suggests a potential role for Slc27a3 in visceral fat accumulation. While further mechanistic investigation is required, we propose that the upregulation of *Slc27a3* in VAT, as observed in our study, may represent an early adaptation event in response to CD feeding, triggering enhanced fatty acid uptake in order to compensate for the increased availability of circulating dietary triglycerides. The observation that this gene was not regulated by CD in SAT is in agreement with other studies showing depot-specific differences in fatty acid influx in obese humans with metabolic syndrome or T2D [32–34].

Epigenetic modifications represent a mechanism through which genetic and environmental exposures impact on the susceptibility to obesity and metabolic diseases [35]. Indeed, DNA methylation alterations have been identified in both human and rodent obesity [28,36], and there is accumulating evidence to show that exposure to high calorie diets, which promote expression and enzymatic activities of DNA methyltransferases, impacts on global and gene-specific DNA methylation [27,28,37,38]. Using an ELISA-based method, we showed that CD induced global hypomethylation in both SAT and VAT. These findings are in line with studies reporting reduced DNA methylation in inguinal (SAT) and epididymal (VAT) adipose tissues from both genetic and dietary obesity mouse models [39]. Moreover, in severely obese humans, Turcot et al. [40] reported that lower global DNA methylation, as assessed by LINE-1 repetitive elements methylation analysis, in VAT is significantly associated with a greater risk for metabolic syndrome. These studies, together with ours, highlight the potential use of epigenetic changes for therapeutic targets for prevention and early treatment of obesity.

Pyrosequencing analysis of *Slc27a3* showed that the CD induced hypomethylation of one CpG site situated 668 bp downstream from the TSS in VAT, which corresponded with increased expression of *Slc27a3* in this depot. While we acknowledge that DNA methylation changes in a single CpG site in response to CD may appear subtle compared to those reported in other obesity-related genes [10,27,41,42], it has been previously demonstrated that even small methylation changes can alter gene expression with significant effects on phenotype. For example, Barrès et al. [11] demonstrated that methylation of a single cytosine residue at the Peroxisome proliferator-activated receptor γ coactivator 1 α (*PGC1α*) promoter is sufficient to reduce gene activity in luciferase reporter assays and can also modulate *PGC1α* mRNA expression. Furthermore, while we note that the CpG affected by the CD in our study is not situated in the upstream promoter region of *Slc27a3*, it has been suggested that DNA methylation downstream of the TSS, particularly in the region of the first exon, is much more tightly linked to transcriptional regulation compared to methylation upstream of the promoter [43]. Based on this, we propose that DNA hypomethylation of the above identified CpG site may play a functional role in the epigenetic upregulation of *Slc27a3* expression in VAT in response to CD feeding, although additional mechanistic studies are required to confirm this.

*In silico* analysis of the *Slc27a3* gene sequence revealed putative transcription factor binding sites for NKX2-1, E2F1, WT1 I and GCF in the region harbouring the six CpG sites investigated in this study. These transcription factors have previously been suggested to play a role in energy expenditure [44], glucose homeostasis [45], adipogenesis [45], insulin secretion [45], lipolysis and β-oxidation [45], control of cell growth [46] and response to hypocaloric dietary intervention [47]. Importantly, the consensus binding site for GCF spans the region harbouring CpG5, which was shown to be hypomethylated in response to CD-feeding in our study. GCF has previously been shown to bind to the GC-rich sequences within the epidermal growth factor receptor (EGFR), β-actin and calcium-dependent protease gene promoters and consequently alter their transcriptional activity [48,49]. Similarly, we propose that CD-induced hypomethylation of *Slc27a3* leads to epigenetic activation of the gene, possibly through increased GCF binding at this region. Future studies should experimentally determine whether GCF binds and regulates *Slc27a3* transcriptional activity through CpG5 and whether this regulation is altered upon DNA methylation changes induced by CD-feeding. It would also be interesting to determine whether *Slc27a3* is transcriptionally activated by WT1, which has been associated with visceral white adipose tissue identity [50]. Addressing these questions would require a combination of mechanistic studies including luciferase reporter gene assays as well as *in vitro* and *in vivo* binding assays such as electrophoretic mobility shift assays (EMSA) and chromatin immunoprecipitation assays, respectively.

Some limitations to this study exist. Due to the cross-sectional study design, we could not determine whether the DNA methylation and/or gene expression signatures identified in this study played a causal role in the different expansion properties observed in VAT and SAT in response to CD, and thus future longitudinal studies are warranted to investigate this. Moreover, the initial PCR array experiments in our study revealed significant downregulation of *Slc27a3* in SAT from CD-fed rats compared to controls, however this was conflicting with our Taqman qRT-PCR data. This may be due to differences in the sample size used for the PCR arrays (n = 8) compared to the qRT-PCR experiments (n = 10) or alternatively, as a result of the different methods employed to quantify gene expression (i.e. SYBR Green dye for the PCR arrays and TaqMan fluorescent probes for qRT-PCR). It is important to note that SYBR Green dye is non-specific and can generate false positive signals if non-specific products or primer-dimers are present in the assay [51]. Thus, PCR array results should be interpreted with caution and validated using alternative gene quantification methods. It is well-established that VAT expansion is accompanied by infiltration of macrophages [52]. Since adipose tissue and immune cells have distinct DNA methylation and gene expression profiles, the infiltration of macrophages could have contributed to the observed differential DNA methylation and gene expression patterns in VAT. Future studies should exclude these potentially confounding effects by quantifying crown-like structure formation within VAT and correcting for these using statistical models as described by Zwamborn et al [28]. We were unable to determine whole-body SAT mass in CD versus STD-fed rats and thus future studies should include the measurement of total fat mass using dual energy X-ray absorptiometry (DEXA). We were also unable to determine *Slc27a3* protein levels in response to CD and STD feeding in the current study and this forms part of future work. Lastly, the molecular function of Slc27a3 in fatty acid uptake and metabolism in VAT during the progression of obesity remains to be elucidated. Gene knockout adipocyte cell culture models may shed light on this and forms part of future studies.

**Figure 5.**
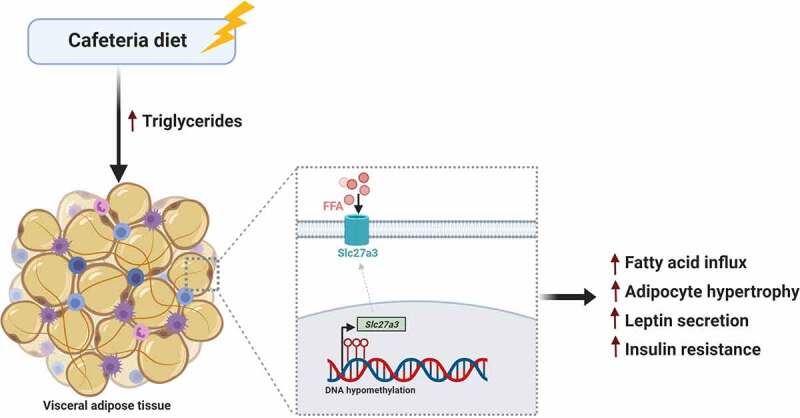
Schematic proposed model of study findings showing how CD leads to adipocyte hypertrophy in VAT, possibly through increased fatty acid influx via epigenetic upregulation of the long chain fatty acid transporter, *Slc27a3*. Increased hypertrophy in VAT is accompanied by increased leptin secretion and insulin resistance. This figure was created using BioRender (https://biorender.com/)

In conclusion, the current study demonstrated that CD exposure led to dynamic changes in DNA methylation and expression of the fatty acid transporter, *Slc27a3*, in VAT. We propose that increased expression of *Slc27a3* by CD in VAT reflects a greater capacity to flux fatty acids across the plasma membrane, leading to adipocyte hypertrophy and insulin resistance (Figure 5). The identification of depot-specific molecular signatures relating to fatty acid uptake may aid in unravelling the putatively adverse metabolic profiles of VAT that contribute to obesity-induced metabolic imbalance and may aid in identifying potential therapies for metabolic diseases.

## Supplementary Material

Supplemental Material
