# Supplementary figures and images for: Cafeteria diet induces global and Slc27a3-specific hypomethylation in male Wistar rats
Source: Adipocyte. 2021 Feb 11;10(1):108–18. doi: 10.1080/21623945.2021.1886697 (PMC7889207; doi:10.1080/21623945.2021.1886697)

Supplementary Figure 1

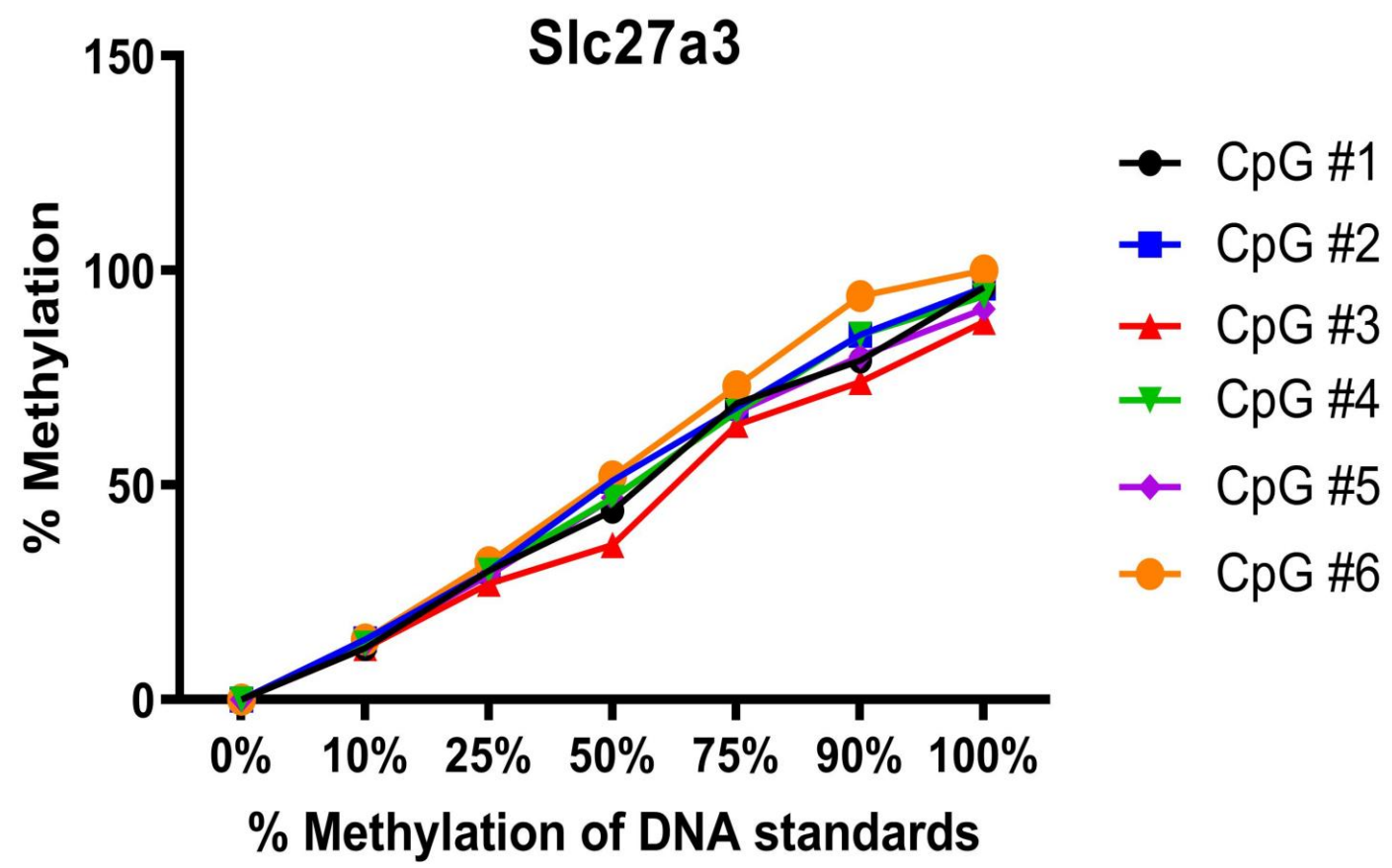

**A**

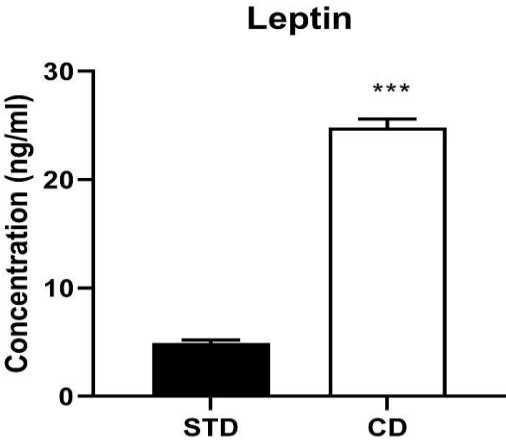

**B**

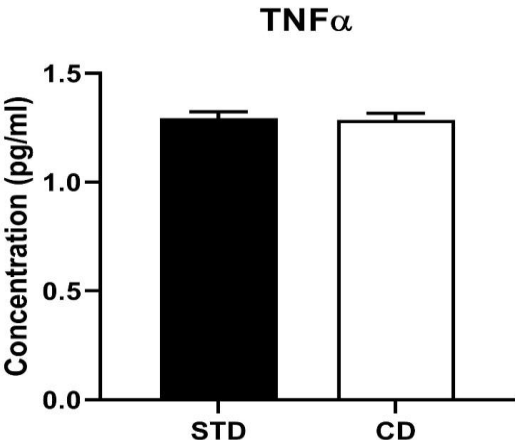

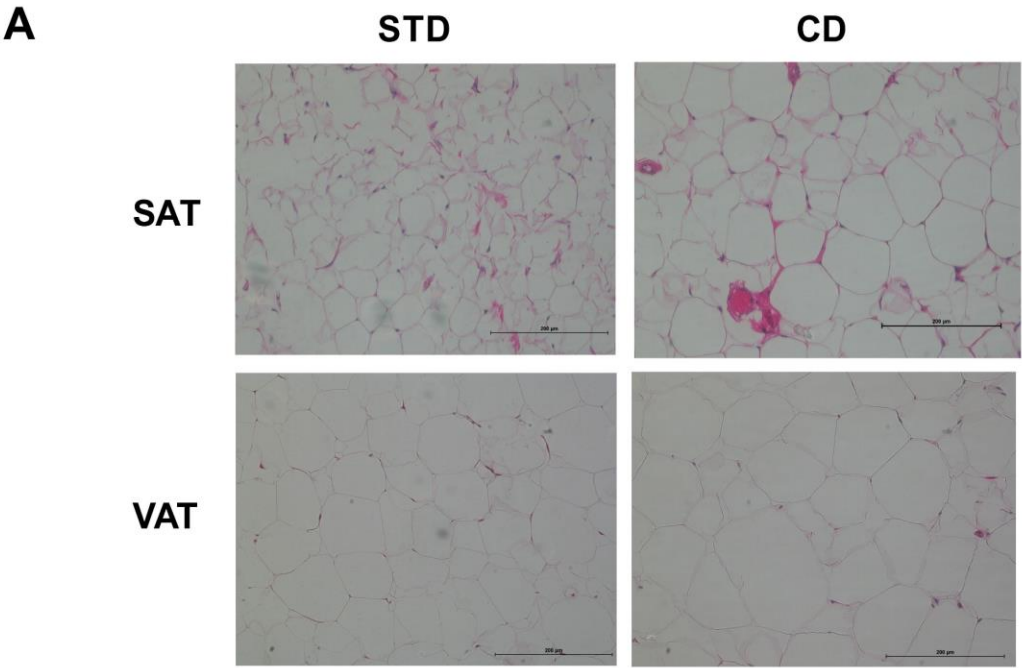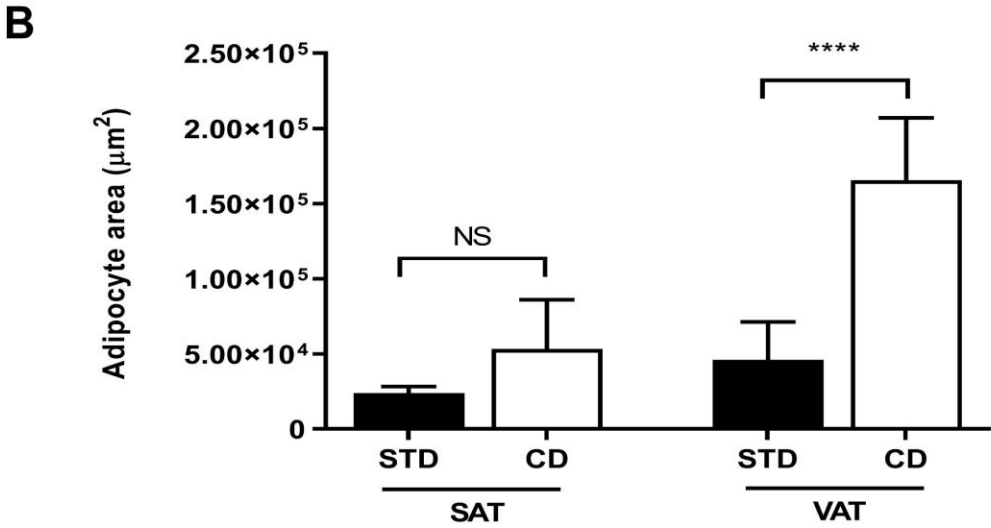

Supplement: Supplemental Material [file KADI_A_1886697_SM1211.pdf]
